# Ulcers of the Cervix Uteri

**Published:** 1871-09

**Authors:** 


### Ulcers of the Cervix Uteri.

M. Despres, surgeon to the Paris hospital for women affected with venereal diseases, has published a book on the above subject. The work has been succintly analysed in the *Montpellier Med.*, April and May, 1871, and the reviewer quotes the following passage, which is worth attention:—"The diseases of women have often proved a rich mine to certain authors, and the extensive practice derived from their writings has mostly been founded on the use of some new agent, hence the many remedies which have been proposed. On the other hand, we find unbiased men giving an account in their works of the therapeutical means which have been extolled, registering failures and occasional success. I am anxious to state my belief that the latter depends mostly on coincidences, and that the cures might have been obtained by other means. It should be remembered that most specialists in the diseases of women use but one mode of cure; such as the red-hot iron, lunar caustic, and more or less concentrated solutions of the latter; all, however, curing ulcers of the cervix in about the same lapse of time." M. Despres ignores all internal remedies, and relies on topical applications. He considers, however, that complete continence, cleanliness, an occasional cauterization by any agent, are very efficacious, especially when patients are treated early, and subjected to constant control. The author adds that he has found the *plug* very useful, and gives the following description of it:—Take a small piece of coarse gause, in which a pledget of cotton wool is placed after it has been filled with about 15 grains of powdered alum. Fold the gauze over the wool, and tie the ends of the former with a thread five or six inches long, which is allowed to hang out of the vagina to facilitate the removal of the plug. The latter should remain twenty-four hours.—*London Lancet.*

